# Ocular findings in 22q11.2 deletion syndrome: A systematic literature review and results of a Dutch multicenter study

**DOI:** 10.1002/ajmg.a.62556

**Published:** 2021-11-12

**Authors:** Emma N. M. M. von Scheibler, Emy S. van der Valk Bouman, Myrthe A. Nuijts, Noël J. C. Bauer, Tos T. J. M. Berendschot, Pit Vermeltfoort, Levinus A. Bok, Agnies M. van Eeghen, Michiel L. Houben, Thérèse A. M. J. van Amelsvoort, Erik Boot, Michelle B. van Egmond‐Ebbeling

**Affiliations:** ^1^ Advisium, 's Heeren Loo Zorggroep Amersfoort The Netherlands; ^2^ Department of Psychiatry and Neuropsychology Maastricht University Maastricht The Netherlands; ^3^ Department of Ophthalmology University Medical Center Utrecht Utrecht The Netherlands; ^4^ University Eye Clinic Maastricht Maastricht University Medical Center Maastricht The Netherlands; ^5^ Department of Ophthalmology Máxima Medical Center Veldhoven The Netherlands; ^6^ Department of Pediatrics Máxima Medical Center Veldhoven The Netherlands; ^7^ ENCORE, Erasmus Medical Center Rotterdam The Netherlands; ^8^ Emma Children's Hospital University of Amsterdam Amsterdam The Netherlands; ^9^ Department of Pediatrics, Wilhelmina Children's Hospital University Medical Center Utrecht Utrecht The Netherlands; ^10^ The Dalglish Family 22q Clinic University Health Network Toronto Ontario Canada

**Keywords:** 22q11.2 deletion syndrome, CNV, cross‐sectional study, ophthalmology, systematic review

## Abstract

The 22q11.2 deletion syndrome (22q11.2DS) is a multisystem disorder with an estimated prevalence of 1:3000 live births. Manifestations show a marked variability in expression and include speech‐ and language delay, intellectual disability, and neuropsychiatric disorders. We aim to provide an overview of ocular findings in 22q11.2DS in order to optimize recommendations for ophthalmic screening. We combined results from a systematic literature review with results from a multicenter cross‐sectional study of patients with 22q11.2DS who were assessed by an ophthalmologist. Our systematic literature search yielded four articles, describing 270 patients. We included 132 patients in our cross‐sectional study (median age 8.9 [range 0–56] years). Most reported ocular findings were retinal vascular tortuosity (32%–78%), posterior embryotoxon (22%–50%), eye lid hooding (20%–67%), strabismus (12%–36%), amblyopia (2%–11%), ptosis (4%–6%), and refractive errors, of which hyperopia (6%–48%) and astigmatism (3%–23%) were most common. Visual acuity was (near) normal in most patients (91%–94%). Refractive errors, strabismus, and amblyopia are treatable conditions that are frequently present in patients with 22q11.2DS and should be corrected at an early stage. Therefore, in 22q11.2DS, we recommend ophthalmic and orthoptic screening at the age of 3 years or at diagnosis, and a low‐threshold referral in adults.

## 1 INTRODUCTION

The 22q11.2 deletion syndrome (22q11.2DS) is a multisystem disorder with an estimated prevalence of 1 in 3000 live births (McDonald‐McGinn et al., 2015). Patients show a marked variability in the clinical expression. Well‐known manifestations include speech‐language and developmental delay, intellectual disability, and an increased risk of developing psychiatric disorders such as schizophrenia and anxiety disorders (Bassett et al., 2005; Campbell et al., 2018; Schneider et al., 2014). Sensory dysfunction has been described as well. For example, hearing loss is frequently reported in 22q11.2DS and large deficits in olfactory function have been described in several studies (Moberg et al., 2020; Verheij et al., 2017). A number of studies have reported on ocular findings in 22q11.2DS, mainly focusing on children (Casteels et al., 2008; Forbes et al., 2007; Gokturk et al., 2016).

The aim of this study is to provide a systematic review of the literature on ocular findings in patients with 22q11.2DS and to present the results of a Dutch multicenter cross‐sectional study of children and adults with 22q11.2DS in order to provide recommendations for ophthalmic screening in 22q11.2DS.

## 2 PATIENTS AND METHODS

### 2.1 Systematic review of the literature

#### 2.1.1 Search strategy and study selection

On January 14th, 2021, we performed a systematic literature search in PubMed, Embase, and Cochrane medical databases (see Supporting Information Material 1 for details). After removing duplicates, titles and abstracts were independently screened by two reviewers (E.V., M. N., and/or E. V. S.). Subsequently, full‐text articles were assessed for eligibility by the three reviewers. All studies that reported on ocular findings, that were assessed by a physician specialized in ophthalmology, in patients with 22q11.2DS were included. We excluded research reporting on patients with a clinical diagnosis of 22q11.2DS, velocardiofacial syndrome, or DiGeorge syndrome, that lacked molecular confirmation. We excluded studies that did not provide prevalences for specific ocular findings. Reviews, case studies, conference abstracts, and non‐human studies were excluded. Discrepancies between authors were resolved by discussion. Reference lists of the included studies were hand‐searched for additional relevant articles.[Boxed-text ajmga62556-fea-0001]

BOX 1 **Ocular concepts and definitions**

**Table:** 

Retinal vascular tortuosity	Abnormal curvature of the retinal blood vessels
Posterior embryotoxon	Corneal abnormality with a thickened and anteriorly displaces Schwalbe's line
Distichiasis	Eyelashes that arise from an abnormal part of the eye lid
Against‐the‐rule astigmatism	Occurs when the horizontal meridian of the cornea is steeper than the vertical meridian
With‐the‐rule astigmatism	Occurs when the vertical meridian of the cornea is steeper than the horizontal meridian
Dacryostenosis	Tear duct obstruction
Dacryocystorhinostomy	Surgical intervention to restore tear flow
Keratoconus	Cone shaped cornea caused by thinning of the cornea
Peters' anomaly	Corneal opacity due to anterior segment dysgenesis

#### 2.1.2 Quality assessment

To assess the relevance and validity of the included articles, we performed a critical appraisal using the Risk of Bias Assessment tool for prevalence studies (Hoy et al., 2012), which was adapted and specified to our research question (see Supporting Information Material 2). The quality of the studies was assessed independently by two reviewers (E. V., M. N. and/or E. V. S.) and discrepancies were resolved by discussion. In the absence of reference scores, we decided to exclude studies with a very high risk of bias (≥7/10 points) for data extraction. Risk of bias assessment included selection bias, standardization, measurement bias, and nonresponse bias. In case of overlap of populations of the same research group, the study with the lowest risk of bias was included.

#### 2.1.3 Data extraction

Data on visual acuity (VA), refractive errors, eye position and motility, eye lid abnormalities, biomicroscopic, and fundoscopic results were extracted by one reviewer and verified by a second reviewer. VA measurements were transformed to logarithm of the minimum angle of resolution (LogMAR) for uniformity. We categorized VA as (near) normal (≤0.30 LogMAR), mild (>0.30 to <0.50 LogMAR), moderate (≥0.50 to <1.0 LogMAR), or severely impaired (≥1.0 LogMAR) according to criteria of the World Health organization (WHO, 2018).

### 2.2 Dutch multicenter cross‐sectional study

#### 2.2.1 Study design and setting

The study was approved by the institutional ethical committees of the University Medical Center Utrecht (UMCU, #18‐510/C), Máxima Medical Center Veldhoven (MMCV, #L20.044), and Maastricht University Medical Center+ (MUMC+, #2019–1321). Through a review of medical records, we systematically compiled data of 22q11.2DS patients that visited the ophthalmological outpatient clinic of UMCU, MMCV, and/or MUMC+ between January 1992 and January 2021. All centers are multidisciplinary outpatient clinics for 22q11.2DS. Ophthalmic screening was carried out as regular screening after diagnosis or referral in all clinics and only in a minority of cases upon clinical indication.

#### 2.2.2 Study subjects

We included patients with a genetically confirmed 22q11.2 deletion. Atypical 22q11.2 deletions were excluded, that is not involving the A‐B region (McDonald‐McGinn et al., 2015).

#### 2.2.3 Data collection

Data on demographic and clinical characteristics included molecular test results, sex, age at most recent ophthalmic screening, reason for referral, congenital heart defects, ophthalmological abnormalities, presence of a headache, prescription of glasses and treatment and/or ocular surgery in the past, and results of most recent ophthalmic screening. Prevalence rates of vascular tortuosity, posterior embryotoxon, and optic disk abnormalities are based on the total number of patients who were examined using fundoscopy and slit lamp.

Best corrected visual acuity measurements were transformed to LogMAR and categorized as described above (WHO, 2018). Spherical refractive errors were divided into six groups comparable to previous studies on ocular findings in 22q11.2DS (Forbes et al., 2007; Gokturk et al., 2016). Refractive errors, myopia and hyperopia, were considered mild in case of more than 0.5 diopters (D) to 2.0D, moderately severe in case of >2.0D and   <4.0D, and severe in case of ≥4.0D. Finally, astigmatism with cylindrical errors of ≤−2.0D were extracted and considered high. Astigmatism was classified as with‐the‐rule, against‐the‐rule, and oblique as described before (Núñez et al., 2019).

### 2.3 Statistical analysis

Categorical data are presented as frequencies with percentage (%) and continuous data are presented as median with ranges. For prevalence rates in our cross‐sectional study, 95% confidence intervals were calculated. We used Spearman's Rank‐Order correlation for studying the degree of association between age and refractive errors, given the asymmetric data distribution. We used *χ* ^2^ tests to compare ophthalmic findings, such as retinal vascular tortuosity, between men and women and between those with and without congenital heart defects. All analyses were two tailed, with statistical significance defined as *p* < 0.05, using IBM SPSS software (Statistics 25; SPSS, Inc.).

## 3 RESULTS

### 3.1 Systematic review of the literature

#### 3.1.1 Study selection

**FIGURE 1:**
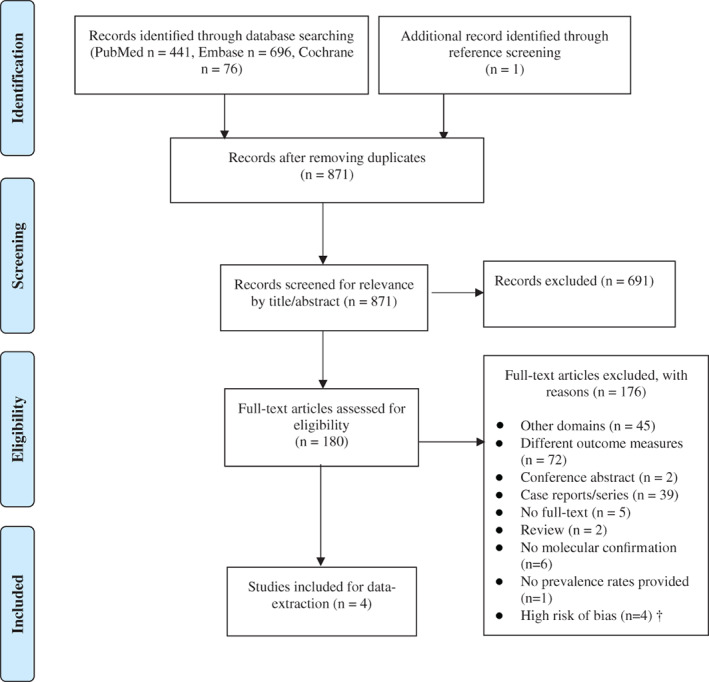
Flow diagram depicting the different phases of the systematic review on ocular findings in patients with 22q11.2DS (adapted from the PRISMA 2009 flow diagram; http://prisma‐statement.org/). ^†^For the complete list, see Supporting Information Material 3

The flow diagram in Figure 1 shows the study selection process. We identified 1213 records through a literature search in PubMed, Embase, and the Cochrane Library. After deduplicating, we screened 871 titles and abstracts for relevance, resulting in 180 articles of which full‐text was screened for eligibility. One article was added after manual search of the reference sections (Ryan et al., 1997). Four studies, including a total of 270 patients, were included for data‐extraction in this systematic review. Four studies were excluded because of a high risk of bias (Supporting Information Material 3) (Cirillo et al., 2014; Ryan et al., 1997; Veerapandiyan et al., 2011; Vieira et al., 2015).

#### 3.1.2 Study characteristics

Table 1 shows the study characteristics of the included studies, all published between 2007 and 2016. Three were prospective cohort studies (Casteels et al., 2008; Forbes et al., 2007; Gokturk et al., 2016), and one was a retrospective cohort study (Midbari Kufert et al., 2016). Median number of included patients was 63 (range 16–128).

**TABLE 1:** Literature review of ocular findings in patients with 22q11.2DS^a^

Author and year	Study design	Country	Patient number	Age (y)	Age range (y)	Retinal tortuosity (%)	Posterior embryotoxon (%)	Optic disk abnormalities (%)	Strabismus (%)	Amblyopia (%)	Ptosis (%)	Eye lid hooding (%)	Hyperopia (%)	Myopia (<2D, %)	Astigmatism (≥2.0D, %)
Midbari Kufert et al., 2016	Retrospective cohort	Israel	128	13(11)	1–55	4	NR	NR	12	2	NR	NR	2^b^	18^b^	2^b^
Forbes et al., 2007	Prospective cohort	USA	90	9	3wk‐37	34	49	1	18	4	4	20	(>2D) 28	4	12
Casteels et al., 2008	Prospective cohort	Belgium	36	7	3–14	78	41	0	36	6	NR	67	(≥2D) 48	10	6
Gokturk et al., 2016	Prospective cohort	Turkey	16	M:7	4 m‐18	56	50	6	25	NR	6	50	(>2D) 6	3	3

*Note*: Prevalences rates were based on the total number of patients except for the prevalence of refractive errors which was based on the number of eyes examined.

Abbreviations: CD, cannot determine because cylinder error was reported for 2 cases only (both >1D and less than 2D); D, diopters; M, median; m, months; NR, not reported; USA, United States of America; wk, weeks; y, year.

^a^ Ages are reported as mean (SD), unless indicated otherwise.

^b^ Cut‐off values for refractive errors were not defined.

In three studies, ophthalmic assessment was done by an ophthalmologist (Casteels et al., 2008; Forbes et al., 2007; Gokturk et al., 2016), and in one study by a subspecialized pediatrician (Midbari Kufert et al., 2016). In this study, additional data were collected through a medical and developmental checklist that was completed by the patient's parents (Midbari Kufert et al., 2016). Two studies reported only on children (Casteels et al., 2008; Gokturk et al., 2016), and two other included mainly children (Forbes et al., 2007; Midbari Kufert et al., 2016). All studies included patients with a 22q11.2 deletion confirmed by fluorescence in situ hybridization test and/or multiplex ligation‐dependent probe amplification technique (Casteels et al., 2008; Forbes et al., 2007; Gokturk et al., 2016; Midbari Kufert et al., 2016).

#### 3.1.3 Quality assessment

Substantial heterogeneity was present among studies concerning demographics, methods, definitions, and outcome measures. The methodology of the studies was poorly described or missing in most studies, complicating comparative evaluation.

#### 3.1.4 Ocular findings

VA was (near) normal in most patients (91%–94%). In one patient, one eye was severely impaired because of a Peters' anomaly (Casteels et al., 2008). One study described that VA was “lower than normal” in two eyes with high hyperopia and one eye with exotropia and high myopia (Gokturk et al., 2016).

Refractive errors were frequently reported in all included studies (Table 1). Hyperopia was the most common refractive error, with a prevalence ranging from 6% to 48% for moderate to severe hyperopia. Moderate to severe myopia (3%–10%) and high astigmatism (3%–12%) were less frequent. One study showed an increase in high astigmatism with age (Forbes et al., 2007).

Ocular findings that were most frequently reported were retinal vascular tortuosity (4%–78%), posterior embryotoxon (41%–50%), strabismus (12%–36%), amblyopia (2%–6%), and optic disc abnormalities (0%–6%) (Table 1). Optic disk abnormalities consisted of hypoplastic or small optic discs (6%), and tilted optic discs (1%). Other ophthalmic findings that were reported were ptosis (4%–6%), distichiasis (2%–6%) (Forbes et al., 2007; Gokturk et al., 2016), lens opacities (3%–6%) (Casteels et al., 2008; Gokturk et al., 2016), glaucoma (6%) (Gokturk et al., 2016), cataract (3%) (Casteels et al., 2008), iriscoloboma (3%) (Casteels et al., 2008), Peters' anomaly (3%) (Casteels et al., 2008), and keratoconus (1%) (Midbari Kufert et al., 2016).

### 3.2 Dutch multicenter study

#### 3.2.1 Results

In our cross‐sectional study, 132 patients (60 males, [45%]) were included. Median age at last ophthalmic screening was 8.9 (range 0–56) years, with 23% aged 18 years or older. Twenty‐two patients (17%) were referred to the ophthalmologist for: suspected visual impairment (*n* = 9), suspected reduced color vision (*n* = 1), persistent conjunctivitis (*n* = 1), recurrent eye lid infection (*n* = 2), entropion (*n* = 1), vitreous floaters (*n* = 1), suspected papilledema (*n* = 1), suspected eye movement disorder (*n* = 1), suspected amblyopia (*n* = 1), strabismus (*n* = 2), and two patients for a second opinion because of esotropia (*n* = 1) and amblyopia (*n* = 1). Five patients received ophthalmic screening because of another underlying condition (juvenile idiopathic arthritis [*n* = 4], diabetes mellitus [*n* = 1]).

VA was available for 109 patients (83%) and was normal in the majority (*N* = 101, [93%]). Of the 23 patients who had no quantitative VA measurement, nine patients (39%) showed good fixation during ophthalmic screening. Reasons for a mild visual impairment (*N* = 5, [5%]) were bilateral or unilateral amblyopia, mild cataract, and high myopia. Three of these five patients (60%) were adults. Moderate visual impairment was found in two children (2%) with only dacryostenosis in one and mild hypoplastic optic disks in the other patient. Both patients were reported to have difficulties with performing the test. One child, with consanguine parents, had a VA of 2.5 logMAR probably caused by keratoconus, subcapsular cataract, and tapetoretinal degeneration. One of the siblings of this patient also had tapetoretinal degeneration. This was the only patient referred to the ophthalmologist for suspected visual impairment in whom visual impairment was found.

Refractive measurements were available for 212 eyes of 106 patients (80%) (Table 2). Moderate to severe hyperopia was seen in 87 eyes (41%) and was persistently high in children from the age of 6 years up to adulthood. Moderate to severe myopia was present in only a small number of eyes (*N* = 12 eyes, [6%]). High astigmatism was reported in 49 eyes (23%). A moderate statistically significant negative correlation was found between age and cylinder power (Spearman's ρ OD: −0.538, *p* = 0.000, OS: −0.510, *p* = 0.000). Most common was against‐the‐rule astigmatism (*N* = 57/128 eyes, [45%]). With‐the‐rule astigmatism (*N* = 37/128 eyes, [29%]) and oblique astigmatism (*N* = 34/128 eyes, [27%]) were seen almost equally often. Glasses were prescribed for 35 children (35%) and 23 adults (74%), prior or during last ophthalmic screening. A headache was reported for 32 patients (24%) recently before or during last ophthalmic screening.

**TABLE 2:** Refractive errors found in 212 eyes of 106 patients with 22q11.2DS

	<6y	6–11.9 y	12–17.9 y	≥18 y	Total
Number of eyes examined	56	68	32	56	212
**Spherical equivalent**	%	%	%	%	%
≤−4.0D (severe)	4	0	0	7	3
−4.0D to −2.01D (moderate)	0	1	6	5	3
−2.0 to −0.51D (mild)	2	9	3	18	8
−0.5 to 0.5D	11	7	16	16	12
0.51 to 2.0D (mild)	46	31	22	32	34
2.01 to 4.0D (moderate)	25	29	31	16	25
≥4.0D (severe)	13	25	22	5	16
**Cylindrical error**					
≤−2D (high)	5	24	25	39	23

Abbreviations: D, diopters; y, years.

Most reported ocular findings are shown in Table 3 and include retinal vascular tortuosity (*N* = 38, [32%]), posterior embryotoxon (*N* = 23, [22%]), strabismus (*N =* 16, [12%]), amblyopia (*N =* 15, [11%]) of which 20% were refractive amblyopia, and optic disk abnormalities (*N =* 15, [13%]) such as hyperpigmentation, hypoplastic, small or tilted optic disks, and excavations. Nine patients (7%) had a history of eye surgery, which included strabismus correction (*N* = 3, [2%]), dacryocystorhinostomy (*N* = 3, [2%]), eye lid correction (*N* = 2, [2%]), and entropion correction (*N* = 1, [1%]). All surgeries, except for one eye lid correction, have taken place in childhood. Prevalence rates of ocular findings in patients younger than 18 years at time of examination were in general slightly lower compared to prevalence rates in all patients. However, it should be noted that some of the ocular findings in adults, especially those that typically manifest in childhood such as amblyopia or embryotoxon posterior, are likely to have been present at an earlier age. Less frequent ocular findings were nystagmus (*N* = 2, [2%]), uveitis (*N* = 1, [1%]), cataract (*N* = 1, [1%]), iris remnants (*N* = 1, [1%]), bilateral corneal ectasia (*N* = 1, [1%]), and one patient with keratoconus, subcapsular cataract, and tapetoretinal degeneration. There was no association between retinal vascular tortuosity and the presence of a congenital heart defect (*χ* ^2^ = 2.19, *p* = 0.33). There were no differences in ocular abnormalities between males and females, except for posterior embryotoxon which was significantly more prevalent in women (18/60, [30%]) compared to men (5/46, [11%]) (*χ* ^2^ = 5.61, *p* = 0.02).

**TABLE 3:** Ocular findings in 132 patients with 22q11.2DS

Ocular findings	Number of patients	Percent of patients <18 y [95% CI]^a^	Percent of total [95% CI]^b^
Retinal vascular tortuosity^c^	38/120	24 [19–39]	32 [23–40]
Posterior embryotoxon^c^	23/106	15 [11–30]	22 [14–30]
Strabismus	16/132	10 [5–18]	12 [6–18]
Eye surgery	9/132	6 [2–13]	7 [2–11]
Optic disk abnormalities^c^	15/120	9 [5–18]	13 [7–19]
Amblyopia	15/132	6 [2–13]	11 [6–17]
Epicanthus	11/132	4 [1–10]	8 [4–13]
Ptosis	6/132	3 [1–8]	5 [1–8]
Motility disorder	3/132	0 [0–4]	2 [0–5]
Dacryostenosis	3/132	3 [1–8]	2 [0–5]
Glasses prescribed	58/132	35 [26–45]	44 [35–53]

Abbreviations: CI, confidence interval; y, years.

^a^ Proportion of patients <18 years at time of examination (*n* = 101).

^b^ Proportion of the total sample (*n* = 132).

^c^ For 12 patients no fundoscopy and for 26 patients no slit lamp examination data was available.

## 4 DISCUSSION

The results of our study indicate that ocular findings are frequently present in patients with 22q11.2DS. We report on ocular findings in the largest cohort of 22q11.2DS patients to date.

Importantly, VA was (near) normal in almost all patients. Severe visual impairment was reported for two children, one with Peters' anomaly and one with keratoconus, posterior subcapsular cataract, and tapetoretinal degeneration with a suspected second genetic hit. It is important to detect visual impairment because of its impact on language and communication development and for its negative effect on psychiatric illness such as depression or anxiety (Demmin & Silverstein, 2020; Mosca et al., 2015). Patients with intellectual disabilities and visual impairment may have an atypical presentation, such as self‐injurious behavior or functional deterioration (de Winter et al., 2011). Also, fatigue and headaches are common in 22q11.2DS (Vergaelen et al., 2017), and may be caused by visual impairment in some cases. When measuring VA of patients with 22q11.2DS, cognitive abilities should be taken into account.

We found a high prevalence of moderate to severe hyperopia, especially in children with 22q11.2DS aged 6 years and older, compared to children and adults in the general population and also children with intellectual disabilities (Akinci et al., 2008; Hashemi et al., 2018; Williams et al., 2015). Studies in the general population have shown that emmetropisation takes place during early development resulting in a reduction and stabilization of refractive errors in early teenage years (Harb & Wildsoet, 2019; Read et al., 2007), which was not the case in our cohort. A possible reason for the high prevalence of hyperopia may be that the axial length of the eye is too short relative to the refractive power of the lens or cornea because of a delay in growth. Also, lag in accommodation has been found in children with severe hyperopia in the general population and may have contributed to the high prevalence of moderate to severe hyperopia in our study (Tarczy‐Hornoch, 2012). In addition, the prevalence of astigmatism in children and adults with 22q11.2DS was much higher compared to the general population and compared to adults with intellectual disabilities (Akinci et al., 2008; van Splunder et al., 2003). Also, in our cross‐sectional study, high astigmatism was more frequently present compared to previous studies in 22q11.2DS. This may be explained by a higher inclusion rate of adults in our study, in whom astigmatism was found more often. Against‐the‐rule astigmatism was most common in all age groups in our cohort and can be influenced by a reduction in lid pressure (Read et al., 2007), which may have contributed to the disturbed emmetropisation in our cohort. Eye lid hooding and ptosis were reported in a substantial number of patients with 22q11.2DS (20%–67% and 4%–6% respectively). Myopia was less frequently reported in children with 22q11.2DS compared to the general population, but a similar prevalence was found for adults (Hashemi et al., 2018). Correction of refractive errors in patients with 22q11.2DS at an early stage is important because it can improve reading abilities (Crewther et al., 1998). Also, high refractive errors and anisometropia have been associated with amblyopia (Brown et al., 2000; Mocanu & Horhat, 2018). Strabismus and amblyopia were frequently reported in 22q11.2DS and may have direct clinical consequences. The prevalence of strabismus and amblyopia is higher compared to the general population (12%–36% versus 1%–3% and 2%–11% versus 1%–4%, respectively) (Hashemi et al., 2019; Webber & Wood, 2005; Yekta et al., 2017), but comparable to what has been reported in children with intellectual disabilities (14% for strabismus) (Akinci et al., 2008). This may suggest that these results are not specific for a 22q11.2 deletion. Clinicians treating patients with 22q11.2DS should be aware of the increased prevalence of refractive errors, strabismus, and amblyopia and their influence on VA and language and communication development if not treated correctly (Blair et al., 2021; Catalano, 1990; Pascolini & Mariotti, 2012). Management of amblyopia includes correction of refractive errors or occlusion therapy and intervention preferably takes place as young as possible because of reduced plasticity of the visual cortex after the age of 7 years (Sengpiel, 2014). The management of strabismus also depends upon the etiology and includes surgical and nonsurgical strategies.

The most common ocular finding, though without clinical consequences, in both the systematic review studies and our cross‐sectional study, was retinal vascular tortuosity (32%–78%). There was one study that reported a prevalence of 4% but did not provide additional information regarding measurement method or an explanation for this very low prevalence compared to other 22q11.2DS studies (Midbari Kufert et al., 2016). Retinal vascular tortuosity has a prevalence of 6% in the general population and therefore may be considered as a typical finding in patients with 22q11.2DS (Henkind & Walsh, 1980). Importantly, retinal vascular tortuosity has been associated with other disorders including obstructive sleep apnea (Mohsenin et al., 2013), diabetes mellitus (Sasongko et al., 2011), and schizophrenia (Appaji et al., 2019) in non‐22q11.2DS populations. These disorders are also frequently reported in patients with 22q11.2DS (Kennedy et al., 2014; Schneider et al., 2014; Van et al., 2020; Zinkstok et al., 2019). In accordance with previous studies in 22q11.2DS, we did not find a correlation between retinal vascular tortuosity and cardiac anomalies (Casteels et al., 2008; Gokturk et al., 2016).

Another common finding in 22q11.2DS was posterior embryotoxon (22%–50%), that also has a higher prevalence compared to the general population (7%) (Rennie et al., 2005). As proposed by others, posterior embryotoxon and other anterior segment abnormalities may be a result of defects in migration, proliferation, and differentiation of neural crest cells in an early embryologic stage in 22q11.2DS (Casteels et al., 2008; Gokturk et al., 2016; Mansour et al., 1987). Anterior segment dysgenesis may increase the risk of glaucoma, which was reported only once in the included review studies (Gokturk et al., 2016) and in not a single patient in our cohort. Other findings supporting a role of a 22q11.2 deletion in anterior segment dysgenesis were scarce, including Peter's anomaly, iris remnants, and lens opacities (Casteels et al., 2008; Gokturk et al., 2016).

With advances in clinical genetic testing, ophthalmic screening is no longer important for diagnosing 22q11.2DS. Another reason for screening after birth could be an increased prevalence of congenital cataract because of its impact on the development of the visual system. However, based on our results, we cannot conclude that congenital cataract has a higher prevalence in 22q11.2DS compared to the general population.

We would recommend that children with 22q11.2DS receive screening by an ophthalmologist and orthoptist at the age of 3 years in order to detect and treat strabismus, amblyopia, and refractive errors, which have high prevalences in 22q11.2DS. From the age of 3 years a reliable monocular VA measurement should be possible. Also, detection of amblyopia is important at an early age because of reduced plasticity of the visual cortex after the age of 7 years. Patients diagnosed with 22q11.2DS after the age of 3 years should receive ophthalmic and orthoptic screening at diagnosis with follow‐up as needed.

For young adults with 22q11.2DS, we recommend low‐threshold referral for ophthalmic and orthoptic screening because of a high prevalence of hyperopia and astigmatism. Clues for visual impairment may be headaches, fatigue, behavioral problems, or functional deterioration. We have no reasons to believe that clinically relevant ocular findings in adults with 22q11.2DS and an intellectual disability are much different from what has been reported for adults with intellectual disabilities in general. Consequently, we have no reasons to deviate from the general guidelines for ophthalmic screening in patients with intellectual disabilities recommending regular screening in late‐adulthood (Evenhuis, 1998; van Splunder et al., 2006). Recommendations for monitoring are provided in Table 4.

**TABLE 4:** Recommendations for ophthalmic and orthoptic screening in 22q11.2DS

Assessment	At diagnosis	At 3 years old	At adulthood
Strabismus	✓	✓	
Amblyopia	✓	✓	
Refractive errors	✓	✓	Low threshold
Visual acuity	✓	✓	Low threshold

*Note*: Each ✓ refers to a single assessment with follow‐up as needed. Ophthalmic screening should be done at least once by an ophthalmologist.

Finally, we would recommend ophthalmological consultation and subsequent testing for a suspected second genetic hit in case of a second, possibly genetic, diagnosis such as tapetoretinal degeneration or congenital cataract.

Large prospective studies with standardized ophthalmological examination and long‐term follow‐up in children and adults are necessary to evaluate the frequency of ocular findings and to study associations between ocular findings and age in 22q11.2DS. Future studies may consider measuring the axial length of the eyeball, corneal shape, and accommodation in order to better understand the high prevalence of hyperopia and delay in emmetropisation in children with 22q11.2DS. Also, more research is needed on sensory disorders in general because of their importance for speech‐language and communication development and in the context of psychiatric comorbidities in 22q11.2DS. These studies will be of value for informing guidelines, especially for adults with 22q11.2DS, which will be updated next year.

Strengths of our cross‐sectional study include the relatively large 22q11.2DS sample and systematic examination by a small number of ophthalmologists. There are also some limitations. First, it is important to note that findings may be difficult to compare between studies due to different definitions, measurement techniques, age, and ethnic and racial groups. Prevalences of some variables such as posterior embryotoxon and vascular tortuosity may differ to a certain extent because of subjective assessment. Nevertheless, our results and previous findings all indicate that these ocular findings are more prevalent in 22q11.2DS compared to the general population. Second, the cross‐sectional study with a retrospective study design made it possible that clinicians have not specifically assessed or reported on all variables for patients who visited the outpatient clinic. Also, age of onset of ocular findings was often lacking from medical files or unknown, making it difficult to report prevalence rates of adult‐onset ocular findings. Taking into account that some patients had difficulties with performing the full examination due to noncooperativity or not understanding instructions, prevalence rates may have been underestimated.

Third, there is a risk of selection bias since most participants in our cross‐sectional study and studies included in this review were assessed in tertiary 22q11.2 centers. However, most participants are referred to these tertiary centers for congenital heart defects, speech and language disorders (including velopharyngeal insufficiency), and/or developmental, psychological, or psychiatric problems. Therefore, we do not expect overestimated ophthalmologic prevalences.

## 5 CONCLUSION

Refractive errors, strabismus, and amblyopia are common, clinically relevant, and treatable ocular findings in patients with 22q11.2DS. Clinicians should be aware of these manifestations and the beneficial result of detection and correction at an early age. Therefore, we would recommend standardized ophthalmic and orthoptic screening in children with 22q11.2DS at the age of 3 years or at diagnosis, and a low‐threshold for referral in adults.

## CONFLICT OF INTERESTS

The authors declare no conflict of interests.

## Supporting information


**Supporting Information Material S1**: Search terms for PubMed, Embase, and Cochrane
Supporting Information Material S2: Critical appraisal form
Supporting Information Material S3: Summary of risk of bias of studies on ocular findings in 22q11.2DS that underwent a critical appraisal

## Data Availability

The data that support our findings are available from the corresponding author on reasonable request.
