# Supplementary material for: Ocular findings in 22q11.2 deletion syndrome: A systematic literature review and results of a Dutch multicenter study
Source: Am J Med Genet A. 2021 Nov 12;188(2):569–78. doi: 10.1002/ajmg.a.62556 (PMC9298823; doi:10.1002/ajmg.a.62556)
Supplement: Supplementary file 1 — Supporting Information Material S1: Search terms for PubMed, Embase, and Cochrane Supporting Information Material S2: Critical appraisal form Supporting Information Material S3: Summary of risk of bias of studies on ocular findings in 22q11.2DS that underwent a critical appraisal [file AJMG-188-569-s001.docx]

**Supplementary material**

1. **Search terms for PubMed, Embase and Cochrane**
   1. **PubMed search terms**

|  | Search terms used in Pubmed |
| --- | --- |
| Domain | ((("22q11 Deletion Syndrome" [MeSH] OR 22q11* [tiab] OR del22q* [tiab] OR DiGeorge [tiab] OR di-george [tiab] OR Velocardiofacial [tiab] OR velo-cardio-facial[tiab] OR VCF-syndrome[tiab] OR (Conotruncal[tiab] AND anomal* [tiab] AND face [tiab]) OR CTAF [tiab] OR "Autosomal dominant Opitz" [tiab] OR "opitz G" [tiab] OR G/BBB [tiab] OR GBBB [tiab] OR "G BBB" [tiab] OR sedlackova [tiab] OR Cayler [tiab] OR catch22 [tiab] OR "catch 22" [tiab] OR shprintzen [tiab] OR "thymic aplasia" [tiab] OR 22q11.2 Deletion Syndrome [tiab])))) |
| Outcome | ((((("Eye Diseases"[Mesh] OR "Eye"[Mesh] OR "Ocular Physiological Phenomena"[Mesh] OR refraction [tiab] OR vision [tiab] OR visual [tiab] OR ophthalm* [tiab] OR eye* [tiab] OR conjunctiv* [tiab] OR cornea* [tiab] OR ocular [tiab] OR optic* [tiab] OR orbit* [tiab] OR retina* [tiab] OR sclera* [tiab] OR uvea* [tiab] OR optic-nerve [tiab] OR eyelid* [tiab] OR lacrima* [tiab] OR lens* [tiab] OR pupil* [tiab] OR iris [tiab] OR intra-ocular [tiab] OR intraocular[tiab] OR cataract [tiab] OR blindness [tiab] OR embryotoxon [tiab] OR tortuous-retinal-veins [tiab] OR retinal-vascular-tortuosity [tiab] OR tortuous-retinal-vessels [tiab] OR refract* [tiab] OR strabismus [tiab] OR exotropia [tiab] OR esotropia [tiab] OR amblyopia [tiab] OR ptosis [tiab] OR distichiasis [tiab] or astigmati* [tiab])))) |
| Excluding | Animals [Mesh:NoExp] OR "mice"[ti] OR "mouse"[ti] OR rat*[ti] OR "nonhuman"[ti] OR veterin*[ti] OR monkey*[ti] |

- 1. **Embase search terms**

|  | Search terms Embase |
| --- | --- |
| Domain | exp chromosome deletion 22q11/ or exp DiGeorge syndrome/ or exp velocardiofacial syndrome/ or exp opitz syndrome/ or (22q11*).ti,ab. **OR** (del22q*OR digeorge OR 'di george' OR velocardiofacial OR 'velo cardio facial' OR 'vcf syndrom*').ti,ab. **OR** ('conotruncal' AND anomal* AND 'face').ti,ab,kw. OR ('ctaf' OR 'autosomal dominant opitz' OR 'opitz g' OR 'gbbb' OR 'g bbb' OR 'sedlackova' OR 'cayler' OR 'catch22' OR 'catch 22' OR 'shprintzen' OR 'thymic aplasia').ti,ab. |
| Outcome | (exp eye disease/ OR exp eye/ OR exp 'visual system parameters'/ OR exp 'visual system function'/) **OR** (refraction OR vision OR visual OR ophthalm* OR eye* OR conjunctiv* OR cornea* OR ocular OR optic* OR orbit* OR retina* OR sclera* OR uvea* OR 'optic nerve' OR eyelid* OR lacrima* OR lens* OR pupil* OR iris OR 'intra ocular' OR intraocular OR cataract OR blindness OR embryotoxon OR 'tortuous retinal veins' OR 'retinal vascular tortuosity' OR 'tortuous retinal vessels' OR refract* OR strabismus OR exotropia OR esotropia OR amblyopia OR ptosis OR distichiasis OR astigmati*).ti,ab. |
| Excluding | (animal/ or animal experiment/ or animal model/ or nonhuman/) not human/  (mice or mouse or rat? or nonhuman or veterin* or monkey?).ti. |
| Limitations | conference abstract or chapter or conference paper or "conference review" or editorial or erratum or letter or note or "review" |

- 1. **Cochrane search terms**

|  | Search terms used in Cochrane |
| --- | --- |
| Domain | ((refraction OR vision OR visual OR ophthalm* OR eye OR eyes OR conjunctiv* OR cornea* OR ocular OR optic* OR orbit* OR retina* OR sclera* OR uvea* OR optic-nerve OR eyelid* OR lacrima* OR lens* OR pupil* OR iris OR intra-ocular OR intraocular OR cataract OR blindness OR embryotoxon OR tortuous-retinal-veins OR retinal-vascular-tortuosity OR tortuous-retinal-vessels OR refract* OR strabismus OR exotropia OR esotropia OR amblyopia OR ptosis OR distichiasis or astigmati*):ti,ab,kw |
| Outcome | (22q11* OR del22q* OR DiGeorge OR di-george OR Velocardiofacial OR velo-cardio-facial OR VCF-syndrome OR Conotruncal anomal* face OR CTAF OR Autosomal dominant Opitz OR opitz G OR GBBB OR G BBB OR sedlackova OR Cayler OR catch22 OR catch 22 OR shprintzen OR thymic aplasia OR 22q11.2 Deletion Syndrome):ti,ab,kw) |

1. **Critical appraisal form**

| Risk of bias item | Scoring | |
| --- | --- | --- |
| *External validity* | | |
| 1. Was the study’s target population a close representation of the standard 22q11.2DS population? | 1 = yes | 0 = no or unclear description |
| 2. Was the diagnosis genetically confirmed in all patients? | 1 = yes | 0 = no or unclear description |
| 3. Was some form of random selection used to select the sample, or was a census undertaken? | 1 = yes | 0 = no or unclear description |
| 4. Was the response rate for the study ≥ 75% or was an analysis performed that showed no significant difference in relevant demographic characteristics between responders and non-responders? | 1 = yes | 0 = no or unclear description |
| *Internal validity* | | |
| 5. Were data collected directly from the subjects (as opposed to proxy)? | 1 = yes | 0 = no or unclear description |
| 6. Was an acceptable definition of ocular features measured in the study used? | 1 = yes | 0 = no or unclear description |
| 7. Was methods of eye examination described? | 1 = yes | 0 = no or unclear description |
| 8. Was the same mode of data collection used for all subjects? | 1 = yes | 0 = no or unclear description |
| 9. Was the length of the shortest prevalence period for the parameter of interest appropriate? | 1 = yes | 0 = no or unclear description |
| 10. Were the numerator(s) and denominator(s) for the parameter of interest appropriate? | 1 = yes | 1. = no or unclear description |

**Source:** *Risk of bias assessment tool for prevalence studies*, Hoy D, Brooks P, Woolf A, Blyth F, March L, Bain C, et al. Assessing risk of bias in prevalence studies: modification of an existing tool and evidence of interrater agreement. J Clin Epidemiol. 2012;65(9):934-9.

1. **Summary of risk of bias of studies on ocular findings in 22q11.2DS that underwent a critical appraisal.**

| **Study**  **Risk of bias item** | Gokturk et al., 2016 | Cirillo et al., 2014 | Casteels et al., 2008 | Forbes et al., 2007 | Vieira et al., 2015 | Veerapandiyan et al., 2011 | Ryan et al., 1997 | Midbari Kufert et al., 2016 |
| --- | --- | --- | --- | --- | --- | --- | --- | --- |
| 1. Target population a close representation of the national 22q11.2DS population? |  |  |  |  |  |  |  |  |
| 2. Was 22q11.2DS genetically proven in all patients? |  |  |  |  |  |  |  |  |
| 3. Some form of random selection used to select the sample? |  |  |  |  |  |  |  |  |
| 4. Likelihood of non-response bias minimal? |  |  |  |  |  |  |  |  |
| 5. Data collected directly from the subjects? |  |  |  |  |  |  |  |  |
| 6. Acceptable case definition of ocular manifestations used in the study? |  |  |  |  |  |  |  |  |
| 7. Methods of ocular examination described? |  |  |  |  |  |  |  |  |
| 8. Same mode of data collection used for all subjects? |  |  |  |  |  |  |  |  |
| 9. Length of the shortest prevalence period for ocular manifestations appropriate? |  |  |  |  |  |  |  |  |
| 10. Were the numerator and denominator for ocular manifestations appropriate? |  |  |  |  |  |  |  |  |
| Overall risk of bias (out of 10 points) * | 3 | 7 | 1 | 4 | 7 | 7 | 7 | 5 |

Light grey= yes (low risk), dark grey=no/unclear (high risk). *≥7/10 points = very high risk of bias, excluded from data extraction.
